# 382. A Multifaceted Intervention to Reduce Blood Culture Contamination in Medical and Surgical Wards: A Cluster Randomized Controlled Trial

**DOI:** 10.1093/ofid/ofad500.452

**Published:** 2023-11-27

**Authors:** Naruemit Sayabovorn, Piriyaporn Chongtrakool, Siriporn Rachakhom, Supaporn Kaewsomnuck, Rakkan Wejsuwanmanee, Chuthaphorn Khamphimabood, Anupop Jitmuang

**Affiliations:** Faculty of Medicine Siriraj Hospital, Bangkok Noi, Krung Thep, Thailand; Faculty of Medicine Siriraj Hospital, Bangkok Noi, Krung Thep, Thailand; Faculty of Medicine Siriraj Hospital, Mahidol University, Bangkok, Krung Thep, Thailand; Siriraj Hospital, Bangkok Noi, Krung Thep, Thailand; Siriraj Hospital, Bangkok Noi, Krung Thep, Thailand; Siriraj Hospital, Bangkok Noi, Krung Thep, Thailand; Faculty of Medicine Siriraj Hospital, Bangkok Noi, Krung Thep, Thailand

## Abstract

**Background:**

Blood culture (BC) is a standard diagnostic testing of bloodstream infection. Inappropriate blood sampling may lead to BC contamination causing antimicrobial overuse and adverse outcomes. Several individualized interventions during blood sampling are applied to reduce BC contamination. However, the efficacy of a multifaceted intervention applied to nursing staff for reducing the contamination rate has yet to be thoroughly studied.

**Methods:**

From August 2020–January 2021, we conducted a 1:1 cluster-randomized controlled trial in 6 medical and 6 surgery wards. Six wards (3 medical, 3 surgery) as the intervention arm received the multifaceted intervention comprising a multimodal educational program, regular surveillance, and feedback. The other six wards (3 medical, 3 surgery) as the control arm received standard care. Participant nurses responsible for BC collection had to answer the pre- and post-study questionnaires to score their background knowledge on appropriate BC sampling. Clinical data and microbiological results were also collected.

**Results:**

Of 3,630 blood culture bottles, 304 (8.4%) were culture positive, with an equal number of true bacteremia and contaminated bottles in both arms. The pre-study contamination rate was 2.2-2.5%. After applying the interventions, the contamination rate was 2.3% in the intervention arm and 3.2% in the control arm (*p*=0.535). Coagulase-negative staphylococci were the most common contaminants (12.8%). Factors associated with contaminated BC included pulmonary diseases (*p*=0.006), length of hospital stay (*p*=0.004), blood sampling at 7:00-15:00 (*p*=0.001), and delayed time to positivity (23.49 h, *p*< 0.001). In addition, nurses who received the interventions achieved overall scores in post-study questionnaires significantly more than nurses who did not (*p*< 0.001).

**Conclusion:**

The multifaceted intervention can maintain a lower contamination rate throughout the study period. It may be a strategy to improve knowledge of an appropriate BC collection for medical staff. This study demonstrated that comorbidity, length of hospitalization, time of blood sampling, and time to culture positivity likely influenced BC contamination.

**Disclosures:**

**All Authors**: No reported disclosures

